# Antibiotics for COPD exacerbations: does drug or duration matter? A primary care database analysis

**DOI:** 10.1136/bmjresp-2019-000458

**Published:** 2019-09-17

**Authors:** Marie Stolbrink, Laura J Bonnett, John D Blakey

**Affiliations:** 1 Institute of Infection and Global Health, University of Liverpool, Liverpool, UK; 2 Department of Biostatistics, University of Liverpool, Liverpool, UK; 3 Respiratory Medicine, Sir Charles Gairdner Hospital, Perth, Western Australia, Australia; 4 Medical School, Curtin University, Perth, Western Australia, Australia

**Keywords:** anti-bacterial agents, lower respiratory tract infections, treatment failure, chronic obstructive, Pulmonary Disease, primary healthcare

## Abstract

**Introduction:**

Antibiotics are routinely given to people with chronic obstructive pulmonary disease (COPD) presenting with lower respiratory tract infection (LRTI) symptoms in primary care. Population prescribing habits and their consequences have not been well-described.

**Methods:**

We conducted a retrospective analysis of antibiotic prescriptions for non-pneumonic exacerbations of COPD from 2010 to 2015 using the UK primary care Optimum Patient Care Research Database. As a proxy of initial treatment failure, second antibiotic prescriptions for LRTI or all indications within 14 days were the primary and secondary outcomes, respectively. We derived a model for repeat courses using univariable and multivariable logistic regression analysis.

**Results:**

A total of 8.4% of the 9042 incident events received further antibiotics for LRTI, 15.5% further courses for any indication. Amoxicillin and doxycycline were the most common index and second-line drugs, respectively (58.7% and 28.7%), mostly given for 7 days. Index drugs other than amoxicillin, cardiovascular disease, pneumococcal vaccination and more primary care consultations were statistically significantly associated with repeat prescriptions for LRTI (p<0.05). The ORs and 95% CIs were: OR 1.28, 95% CI 1.10 to 1.49; OR 1.37, 95% CI 1.13 to 1.66; OR 1.33, 95% CI 1.14 to 1.55 and OR 1.05, 95% CI 1.02 to 1.07, respectively. Index duration, inhaled steroid use and exacerbation frequency were not statistically significant. The derived model had an area under the curve of 0.61, 95% CI 0.59 to 0.63.

**Discussion:**

The prescription of multiple antibiotic courses for COPD exacerbations was relatively common—one in twelve patients receiving antibiotics for LRTI had a further course within 2 weeks. The findings support the current preference for amoxicillin as index drug within the limitations of this observational study. Further clinical trials to determine best practice in this common clinical situation appear required.

**Key messages**

- A substantial proportion of COPD patients received a second course of antibiotics within fourteen days of the index prescription.
- Amoxicillin was prescribed most commonly as the index drug and was associated with fewer repeat antibiotic prescriptions.
- Shorter index courses were not associated with more repeat prescriptions.

## Introduction

Lower respiratory tract infections (LRTIs) are globally the most common infectious cause of morbidity.^1^ Despite this prevalence, observed duration of antibiotic treatment for LRTI varies greatly, and there is disagreement between guidelines on the optimal duration.^2^ Effective first-line treatment for non-pneumonic LRTI in chronic obstructive pulmonary disease (COPD) patients (‘infective exacerbations’) is an area of particular uncertainty.

COPD is a common cause of disability, and estimated to become the third leading cause of death worldwide in 2030.^3–5^ Exacerbations form a large part of the disease burden and can lead to a cough with discoloured phlegm irrespective of causation. Over £250 million is spent on treating COPD exacerbations annually in the UK, and recurrent exacerbations are associated with increased morbidity and mortality.^6^

Up to half of all COPD exacerbations are thought to be caused by bacteria, the remainder by viruses or environmental irritants.^7–10^ The most common pathogens are *Haemophilus influenza*e, *Moraxella catarrhalis* and *Streptococcus pneumoniae*.^6^ Initial studies suggested that the administration of antibiotics was associated with a lower risk of symptom persistence.^11^ However, a Cochrane review concluded that a statistically significant improvement in treatment failure rate was only seen in severe exacerbations, with more adverse events in the antibiotic group.^7^ Hence, the European Respiratory Society/American Thoracic Society and international guidelines advise, based on moderate evidence, the prescription of antibiotics for ambulatory patients ‘if clinically indicated’.^12 13^ However, our understanding of the success of real-life prescribing practices is limited.

The risk of treatment failure without antibiotics needs to be balanced with antimicrobial resistance, contributed to by inappropriate and non-evidence based prescribing, and adverse drug effects, including *Clostridium difficile* infection.^14–16^ Primary care is an optimal environment to improve antibiotic use since 74% of all UK antibiotics are prescribed here and around three-quarters of patients presenting in primary care with an acute COPD exacerbation receive antibiotics.^17–19^ Moreover, a European-wide COPD audit of hospitalised patients demonstrated that antibiotics were more likely to be continued during admissions and after discharge if they had already been received in primary care.^20^

Guidelines on antibiotics for COPD exacerbations are not specific and based only on moderate evidence. The National Institute of Health and Care Excellence guidelines for the study period suggest using an aminopenicillin, macrolide or tetracycline but give no indication on duration.^21^ The Global Initiative for Chronic Obstructive Lung Disease advises 5–7 days duration but no antibiotic class.^13^ A recent meta-analysis presented dirithromycin, ofloxacin and ciprofloxacin as having the best cure and side-effect profiles, yet these are not used routinely in clinical practice.^22^

In this study, we characterised patterns of antibiotic prescribing for COPD exacerbations via a retrospective observational analysis of primary care data from 2010 to 2015. We explored the factors associated with the risk of further antibiotic prescription, which may form the basis for future comparative interventional studies, such as clinical trials comparing different antibiotic durations, or first-line drugs in specific patient groups.

## Methods

We carried out a cross-sectional database study drawing on retrospective, electronic medical records from the Optimum Patient Care Research Database (OPCRD). Individuals were included if they had an active diagnosis of COPD during the study period, demonstrated by their primary care coding.

We included COPD patients who received at least one antibiotic prescription with an LRTI read code from 01 April 2010 to 01 April 2015. LRTI codes included those for chest infection and bronchitis. We analysed only the first event for each individual to exclude repeated measurements from the same patient. Primary outcome was a new antibiotic prescription with an LRTI code within 14 days of the index prescription. Secondary outcome was further antibiotic prescription within 14 days for any indication. We excluded cases with a coded diagnosis of pneumonia, other chronic respiratory diseases, such as asthma, people younger than 16 years and those whose index antibiotic course was either not specified or was longer than 28 days.

### Data source

The OPCRD comprised data extracted through the Optimum Patient Care Clinical Service Evaluation (http://optimumpatientcare.org/opcrd/). The OPCRD is a quality-controlled, primary care research database focussing on respiratory disease. It contained anonymous, routinely recorded electronic medical records data from over 525 UK general practices.

### Statistical analysis

Data were analysed using SPSS V.22. We examined multiple factors to assess the risk of repeat antibiotic prescription for LRTI or any indication, including demographics, smoking status, comorbidities, COPD disease control, including medication and interactions with secondary care, and index antibiotic duration and class.

Individual factors were first assessed using univariable analysis. Univariably, mean differences in continuous variables were analysed via t-tests, while ORs for binary variables were analysed via logistic regression. Multivariable models were built using logistic regression with backwards selection. Summary statistics, $\chi^{2}$ and Student t tests were presented as appropriate for each variable, based on type and distribution of data. Statistically significant results were defined as p<0.10 for univariable analysis (to show trends) and p<0.05 for multivariable analysis. Collinear associations between clinically plausible-related predictors were assessed using Pearson’s correlation coefficients. Factors were included in the multivariable model based on clinical plausibility. Adjusted ORs and CIs were presented where appropriate. Visual inspection of the functional form of each continuous variable suggested that no transformation was required. Multivariable models were derived via backwards parsimonious logistic regression, including linear regression for mean differences, and the resulting models were tested for goodness of fit.

## Results

### Demographics

There were 22 003 unique prescriptions for LRTI in adult COPD patients between 01 January 2010 and 31 December 2015 in the OPCRD. The mean age was 71 years and 48.2% were women. A total of 45.6% were ex-smokers and 42.4% were current smokers, 4.9% were never-smokers (smoking status was missing in 7.1%). Most patients were from the Midlands and East England (58.9%). The most common LRTI codes were for ‘chest infection’ (44.3%), ‘acute exacerbation of chronic obstructive airways disease’ (28.1%) and ‘lower respiratory tract infection’ (17.3%). Table 1 describes the baseline demographics and all variables included in the univariable analysis.

**Table 1:** Baseline demographics of all individuals included in the study

Variable	All patients (n=22 003) Number (%) unless otherwise indicated
Female sex	10 605 (48.2)
Age (years), mean±SD	70.83±10.9
BMI (kg/m^2^), mean±SD	27.2±6.1
Smoking status	
Non-smoker	1081 (4.9)
Current smoker	9326 (42.4)
Ex-smoker	10 033 (45.6)
Missing	1563 (7.1)
Primary and secondary care consultations	
Number of respiratory consultations in primary care*	
1	4371 (19.9)
2	7823 (35.6)
3	4269 (19.4)
≥4	3445 (25.1)
Number of all consultations in primary care*, mean±SD	12.9±9
Number who had at least one inpatient admission for respiratory code*	326 (1.4)
Exacerbations*	
0	10 203 (46.4)
1	6831 (31.0)
2	2729 (12.4)
≥3	2240 (10.2)
Lung function	
FEV1 (L), mean±SD	1.43±0.61
FEV1/FVC ratio, mean±SD	0.59±0.3
COPD treatment	
Number of SABA prescriptions*, mean±SD	4.78±5.3
Number using ICS	12 925 (58.7)
Number of LAMA prescriptions*, mean±SD	3.02±4.5
Number of LABA inhalers*, mean±SD	0.41±2.0
No treatment	2718 (12.4)
Vaccinations	
Influenza vaccination ever	13 894 (63.1)
Pneumococcal vaccination ever	6860 (31.2)
Blood eosinophil count closest to index antibiotic prescription, mean±SD	0.31±0.5
Recorded comorbidity	
Diabetes	3286 (14.9)
Anxiety/depression	1811 (8.2)
Cardiovascular disease	2407 (10.9)
Concurrent medications	
Paracetamol prescription*	10 554 (48.0)
Location	
London	86 (0.4)
Midlands and East	12 965 (58.9)
North	4577 (20.8)
South	32 349 (14.8)
Missing	1126 (5.1)

*In year prior to index prescription.

BMI, body mass index; FEV1, forced expiratory volume in 1 s; FVC, forced vital capacity; ICS, inhaled corticosteroid; LABA, long-acting beta-agonist; LAMA, long-acting muscarinic antagonist; SABA, short-acting beta-agonist.

**Figure 1:**
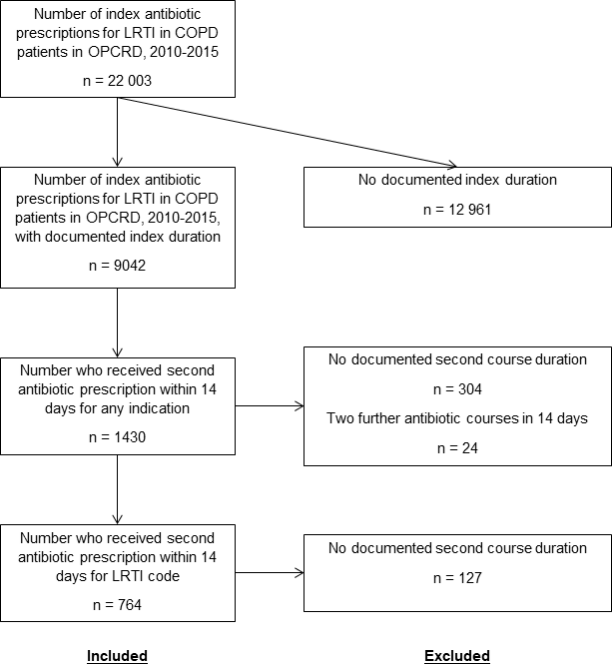
Flow chart of inclusion and exclusion for study analysis of antibiotic prescriptions in COPD patients in the OPCRD database, April 2010–April 2015. COPD, chronic obstructive pulmonary disease; LRTI, lower respiratory tract infection; OPCRD, Optimum Patient Care Research Database.

**Figure 2:**
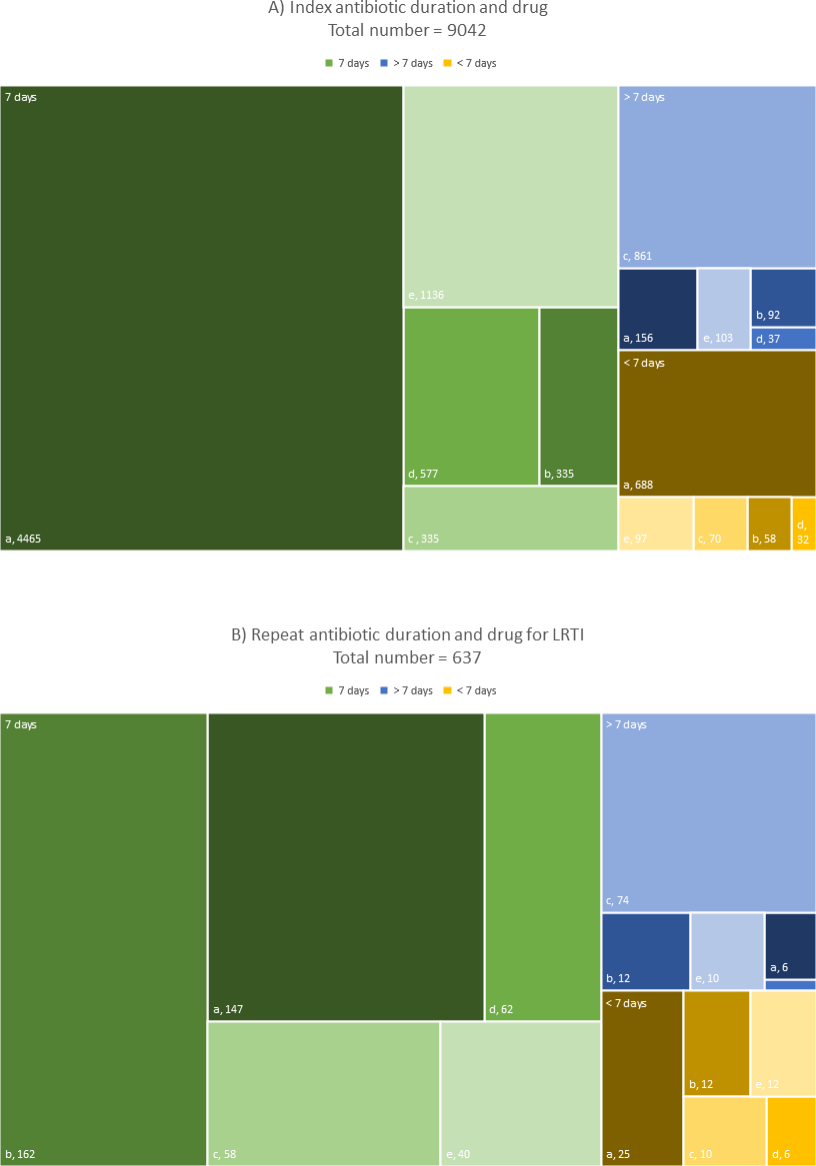
Treemap chart of antibiotic duration and drug of (A) index prescriptions and (B) repeat prescriptions for LRTI. Absolute numbers presented. Missing data due to missing duration (127 cases). a, amoxicillin; b, erythromycin/clarithromycin; c, doxycycline; d, co-amoxiclav; e, other; LRTI, lower respiratory tract infection.

A total of 9042 (41%) patients had a documented index antibiotic duration for LRTI and were included in the analysis (figure 1). Individuals receiving antibiotics for an uncertain duration (eg, only number of tablets documented) were excluded, as were a small number of individuals with treatment durations over 28 days. Most index courses were prescribed for 7 days (75.7%), 10.5% received shorter and 13.8% received longer index courses. Amoxicillin, doxycycline and clarithromycin were used most commonly (58.7%, 14.0% and 11.7% respectively, see figure 2A). The median index duration was 8 days for doxycycline and 7 days for all other agents.

### Repeat prescriptions for LRTI

A total of 764 (8.4%) patients received a second antibiotic course for LRTI within 14 days of the index prescription. The most commonly prescribed antibiotics were doxycycline, erythromycin/clarithromycin and amoxicillin (28.7%, 27.3% and 25.8%, respectively, see figure 2B). Most second-line prescriptions were for 7, 8 or 5 days (73.5%, 9.8% and 8.6%, respectively). The median for repeat antibiotic course duration was 7 days. A total of 127 cases had no documented second-course duration.

In the univariable analysis, those who received further antibiotics were statistically significantly older, had more comorbidities and had more severe disease as measured by higher consultation frequency, more COPD exacerbations in the past year and more COPD medications (table 2). Index drug and location were significantly associated with repeat prescription. Smoking status, FEV1 (forced expiratory volume in 1 s), index duration and ICS (inhaled corticosteroids) treatment were not significantly associated with repeat prescriptions (supplementary files).

10.1136/bmjresp-2019-000458.supp1 Supplementary data

**Table 2:** Results of statistically and clinically significant univariable and multivariable analysis for repeat antibiotic prescription for LRTI code within 14 days of index duration. Univariable analysis according to type of data

Variable	Univariable analysis results			Multiple logistic regression model results	
	OR 95 % CI	Mean difference 95% CI	P value	OR 95 % CI	P value
Age		−0.66 −1.4 to 0.1	0.10		
Location			<0.01		
Midlands and East	Index				
London	0.59 0.21 to 1.62		0.30		
North	0.74 0.60 to 0.91		<0.01		
South	0.77 0.64 to 0.93		0.01		
Gastro-oesophageal reflux disease diagnosis	1.53 1.03 to 2.27		0.03		
Cardiovascular disease diagnosis	1.63 1.34 to 1.95		<0.01	1.37 1.13 to 1.66	<0.01
Paracetamol prescription*	1.18 1.02 to 1.37		0.03		
Pneumococcal vaccination	1.38 1.19 to 1.60		<0.01	1.33 1.14 to 1.55	<0.01
Number of primary care respiratory consultations*		−1.05 −1.29 to 0.82	<0.01	1.05 1.02 to 1.07	<0.01
Number of all primary care consultations*		−3.16 −3.89 to −2.43	<0.01	1.01 1.01 to 1.02	<0.01
Number of OCS*		−0.10 −0.20 to 0.00	0.05		
Number of exacerbations*		−0.10 −0.20 to −0.01	0.06		
FEV1 value (L)		0.02 −0.03 to 0.07	0.38		
Count of ICS inhalers*		−0.41 −0.87 to 0.05	0.08		
Count of LAMA prescriptions*		−0.33 −0.67 to 0.01	0.06		
Count of LABA inhalers*		−0.20 −0.36 to −0.04	0.02	1.03 1.00 to 1.05	0.08
Count of LTRA prescriptions*		0.04 −0.16 to 0.00	0.06		
Not amoxicillin as index antibiotic	1.37 1.18 to 1.58		<0.01	1.28 1.10 to 1.49	<0.01

Blank entries relate to variables not included in the multivariable model.

*In year prior to index prescription.

FEV1, forced expiratory volume in 1 s; ICS, inhaled corticosteroid; LABA, long-acting beta-agonist; LAMA, long-acting muscarinic antagonist; LRTI, lower respiratory tract infection; LTRA, Leukotriene receptor antagonist;OCS, oral corticosteroid.

Collinearity was assessed for all statistically significant (p<0.10) factors in univariable analysis. The Pearson’s correlation coefficient was in excess of 0.8 between the clinically related pairs of number of oral steroid courses and exacerbation number, number of respiratory and all secondary care outpatient appointments, number of ICS inhalers and prescriptions, pure LAMA (long-acting muscarinic antagonist) prescription and inhalers, and pure LABA (long-acting beta-agonist) prescription and inhalers. We, hence, used exacerbation number, respiratory outpatient appointments, ICS, LAMA and LABA inhalers in the multivariable analysis.

The derived parsimonious multivariable model included index antibiotic, number of primary care consultations for respiratory and all complaints, number of LABA inhalers in previous year, presence of cardiovascular disease and previous pneumococcal vaccination (p<0.05, table 2). Those not receiving amoxicillin as index antibiotic, who had previously received the pneumococcal vaccine, with cardiovascular disease, more LABA inhalers or more respiratory or all primary care consultations, were at increased risk of repeat antibiotics for LRTI.

The model fitted the dichotomous outcome with a Cox-Snell R^2^ of 0.013 (area under the curve (AUC) 0.61, 95% CI 0.59 to 0.63). Introducing interaction terms between clinically logical factors did not improve the fit of the model (supplementary files).

### Repeat prescriptions for all indications

A total of 1430 patients received a second antibiotic course for any indication (15.5%). Twenty-four of these received two courses within 14 days of index prescription and were, hence, excluded from further analysis. Doxycycline, amoxicillin and erythromycin/clarithromycin were used most (28.9%, 27.0% and 26.6%, respectively, supplementary figure 1) for durations of 7, 8 and 5 days (71.4%, 9.2% and 8.1%, respectively). The antibiotics included in the ‘all indications’ analysis were amoxicillin, doxycycline, erythromycin, clarithromycin, co-amoxiclav, cephalexin, cefaclor and ciprofloxacin, all of which are used to treat LRTI. The median duration was 7 days. A total of 304 cases did not have a documented repeat duration.

Smoking status, number of emergency department and outpatient appointments, FEV1 as well as ICS treatment were statistically significant (p<0.05) in the univariable analysis (supplementary files).

The presence of cardiovascular disease, index drug other than amoxicillin, number of all primary care consultations and exacerbations as well as number of ICS and LAMA inhalers were significantly associated with repeat antibiotic prescription in the multivariable parsimonious regression analysis (p<0.05). Model-checking showed a Cox-Snell R^2^ of 0.012 (AUC 0.58, 95% CI 0.56 to 0.60).

## Discussion

### Summary

In this analysis of COPD exacerbations in UK primary care, we studied over 9000 antibiotic prescriptions for LRTI over 5 years. The majority of patients received 7-day index courses and amoxicillin was the most commonly given drug. A large proportion of antibiotic prescriptions had no explicitly recorded duration. A substantial proportion of patients (15.5%) received further antibiotics within 14 days of the index antibiotic prescription. Over half of the repeat prescriptions within 14 days were coded for another LRTI (52.1%). Amoxicillin as index drug was associated with fewer repeat prescriptions. Older age and some markers of COPD severity were associated with increased risk of apparent initial treatment failure.

### Strengths and limitations

One of the strengths of the study is the genuine, unselected primary care population as demonstrated, for example, by 97.2% not having had a respiratory outpatient appointment and 46.4% not having had an exacerbation in the prior year. This contrasts with most interventional studies which have highly selected patient populations—for example, it has been estimated that only up to 7% of people treated for COPD meet inclusion criteria for randomised trials.^23^ Real-life data, such as this, have an important role in helping to inform real-life decision-making.

We analysed electronic health records. In the era of advancing electronic records, there is a drive to use more routinely collected data particularly in primary care research, for example, by the National Institute for Health Research or highlighted in the Salford Lung Study, to reduce burden on patients, bias and approximate real-world practice.^24 25^ However, the reliance on primary care data is also this study’s major limitation—chiefly, the 59% of patients for whom no duration was clearly documented for their index duration of antibiotics. This reflects the true documentation practice in primary care, where coding can be challenging.^26^ We are unable to distinguish appropriate from inappropriate antibiotic prescribing using this large primary care database, in keeping with large variation in antibiotic prescribing in primary care found in other studies that could not entirely be explained by patient characteristics.^27^ It is also likely that other factors, such as presence of purulent sputum or prior microbiology results, influenced decisions for repeat antibiotic prescribing. A cross-sectional primary care study in Europe, Asia and South America showed that purulent sputum and use of C reactive protein were the strongest predictors of initial antibiotic prescribing in COPD exacerbations.^18^

We examined more antibiotic courses over 5 years than an earlier primary care COPD exacerbation analysis from 2005 to 2010.^27^ This study of 12 609 exacerbations also showed a high prevalence of antibiotic prescribing within 7 days of presentation (66% cases). We also reported repeat antibiotic prescribing for any indication to ensure no LRTI prescriptions were missed as not all repeat prescriptions for LRTI will have been coded appropriately.

We were limited by the potential for missing data on secondary care contacts (eg, emergency department attendances and hospital admissions) and use of other healthcare services (eg, out of hours and walk-in centres). The imperfect recording of such data is anticipated to lead to an underestimate of the true number of repeat prescriptions issued as lack of improvement increases the probability of accessing providers other than the patient’s usual general practitioner. We acknowledge that some treatment failures would result in hospitalisation, and these could be incorrectly coded as if the treatment was successful (ie, no repeat course of antibiotics from primary care). However, community-treated exacerbations of COPD are far more common than hospitalisations, and most people admitted have not completed antibiotics in the community, so any effect on the analyses would be modest.^20^ There was no explicit coding for repeat or continuous antibiotics in this data set. However, such prescriptions would have been excluded (as we did not include antibiotic courses that had a duration that was either not specified or was longer than 28 days) unless they were rotating antibiotics which are used very rarely, for example, 1 patient in over 92 000 cases studied in a large retrospective primary care analysis.^28^ The use of long-term antibiotics in COPD patients remains rare (eg, 0.61% in the above study) and no long-term studies exist in these patients.^28–30^ Our study is also limited by primary care coding of COPD rather than the use of source data, such as lung function.

### Setting in current literature and future directions

**Figure 3:**
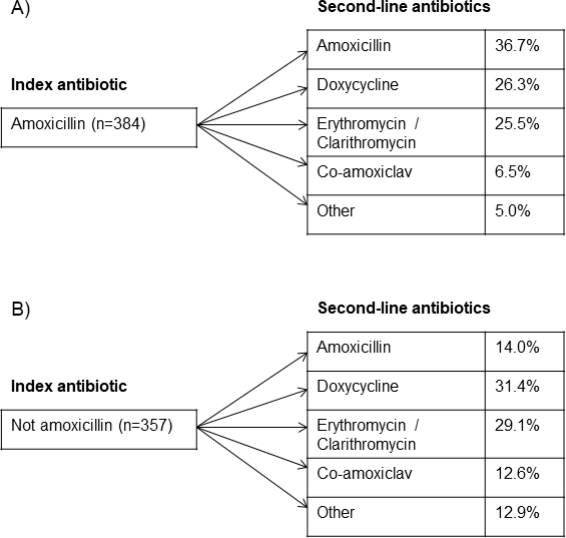
Second-line antibiotics used for LRTI by initial antibiotic: (A) amoxicillin and (B) not amoxicillin. Missing data due to missing drug name (23 cases). LRTI, lower respiratory tract infection.

Durations of 7 days remained most prominent across different situations, but index duration was not significantly associated with further prescriptions. When encountering treatment failure, the decision was to change drug rather than duration (figure 3)—studies are needed to investigate whether this is the correct approach. There is limited evidence and guidance on ideal index duration for antibiotics.^13 21^ A recent meta-analysis found that adverse effects in COPD patients were more prevalent when the same index antibiotic was given for more than 7 days compared with less than 7 days with no difference in clinical outcomes.^31^ A large retrospective analysis of UK primary care data showed that respiratory infections were responsible for the majority of antibiotics prescribed for longer than guidelines suggest, with 89% of COPD exacerbations receiving more than the recommended 5 days of treatment.^32^ In primary care, asthma patients 7-day courses were also associated with fewer repeat prescriptions.^33^ Our study also supports the consideration of shorter index durations, but interventional studies are needed to confirm this.

Prior pneumococcal vaccination was associated with repeat antibiotic prescription. Vaccination did not appear to be a proxy of comorbidity, age or frequency of primary care attendance given its appearance in the final multivariable model, that interaction analysis did not improve the model and there was no collinearity. In previous studies, pneumococcal vaccination has been shown to reduce vaccine-specific disease but not overall rates of LRTI.^34^ It is, therefore, possible that these vaccinated patients may have had a true bacterial infection that was not susceptible to the common index drugs. Owing to the observational, retrospective nature of the analysed data sets, the causality of effects is difficult to ascertain. This is particularly true given a further limitation of our study: sputum culture results were not sufficiently commonly or systematically recorded to be included in the analyses.

ICS have previously shown to be associated with increased risk of pneumonia, particularly older and more severely airway constrained patients.^35–37^ These factors were associated with apparent treatment failure in our univariable analyses, but not in the final models.

Amoxicillin, doxycycline and erythromycin/clarithromycin were the most commonly used antibiotics. The prevalence of amoxicillin decreased from 58.7% to 25.8% in the repeat prescriptions but remained an important contributor. This may reflect practitioners attempting to address relevant pathogens, such as *M. catarrhalis* and *H. influenzae*, in the repeat courses that are usually resistant to penicillins.^38^ However, in contrast to this, not using amoxicillin as index antibiotic was significantly associated with the risk of repeat prescriptions for LRTI and all indications. This warrants further investigation on the pathogens of these exacerbations, particularly to differentiate true failure of antibiotic action from unacceptable side effects from the index treatment. Respiratory viruses were identified in almost 40% of patients with COPD exacerbations and only 40%–50% of exacerbations were thought to be caused by bacteria.^9 10^ Non-bacterial exacerbations would improve irrespective of antibiotic treatment and inappropriately treated exacerbations may present as treatment failure.

## Conclusion

Prescription of further antibiotics for LRTI occurred commonly in UK primary care COPD patients from 2010 to 2015. Our data supported usage of amoxicillin as index drug. Shorter index durations were not associated with more repeat prescriptions. Prior consultations, pneumococcal vaccine, presence of cardiovascular disease and index drug were associated with the decision to prescribe a second course. Interventional studies on the optimal drug and duration for common subsets of people with COPD in primary care are needed to properly inform guidelines for this common clinical problem.
