# Supplementary material for: Antibiotics for COPD exacerbations: does drug or duration matter? A primary care database analysis
Source: BMJ Open Respir Res. 2019 Sep 17;6(1):e000458. doi: 10.1136/bmjresp-2019-000458 (PMC6797433; doi:10.1136/bmjresp-2019-000458)

Supplementary table 1: Results of univariable analysis of all factors for repeat antibiotic prescription for LRTI code. Columns are n (%), unless otherwise stated.

(OR: odds ratio; 95 % CI: 95 % confidence interval; * in year prior to index prescription; #: mean difference)

Variable	**No LRTI antibiotic prescription within 14 days of index prescription N (%)**	**Further LRTI antibiotic prescription within 14 days of index prescription N (%)**	**p value**	**OR 95 % CI**
Gender			0.78	
Female	3987 (91.5)	372 (8.5)		
Male	4291 (91.6)	392 (8.4)		0.98<br>0.84 – 1.14
Age, mean ± SD	70.49 ± 10.6	71.15 ± 10.6	0.10	- 0.66<br>- 1.4 – 0.1
BMI, mean ± SD	27.33 ± 6.1	27.14 ± 5.8	0.39	0.20<br>-0.25 – 0.66
Smoking status			0.25	
Non-smoker	726 (92.1)	62 (7.9)	0.89	0.98<br>0.73 – 1.31
Current smoker	2983 (92.0)	260 (8.0)		
Ex-smoker	4433 (91.0)	437 (9.0)	0.13<br>Chi-square	1.13<br>0.96 – 1.33
Location			0.005<br>Chi-square	
Midlands and East	4025 (90.3)	430 (9.7)		
London	64 (93.9)	4 (6.1)	0.30<br>Chi-square	0.59<br>0.21 – 1.62
North	1631 (92.7)	129 (7.3)	<0.01<br>Chi-square	0.74<br>0.60 – 0.91
South	1940 (92.4)	160 (7.6)	0.01<br>Chi-square	0.77<br>0.64 – 0.93
Recorded co-morbidity				
Eczema diagnosis			0.45<br>Chi-square	
No	6214 (91.7)	564 (8.3)		

Yes	2064 (91.2)	200 (8.8)		1.07<br>0.90 – 1.26
Rhinitis diagnosis			0.05<br>Chi-square	
No	7288 (91.8)	654 (8.2)		
Yes	990 (90.0)	110 (10.0)		1.24<br>1.00 – 1.53
Diabetes diagnosis			0.77<br>Chi-square	
No	7054 (91.6)	648 (8.4)		
Yes	1224 (91.3)	16 (8.7)		1.03<br>0.84 – 1.27
Gastro-oesophageal reflux diagnosis			0.03<br>Chi-square	
No	8070 (91.7)	735 (8.3)		
Yes	208 (87.8)	29 (12.2)		1.53<br>1.03 – 2.27
Anxiety / depression diagnosis			0.21<br>Chi-square	
No	7585 (91.7)	690 (8.3)		
Yes	693 (90.4)	74 (9.6)		1.17<br>0.91 – 1.51
Cardiovascular disease diagnosis			<0.01<br>Chi-square	
No	7072 (92.2)	598 (7.8)		
Yes	1206 (87.9)	166 (12.1)		1.63<br>1.34 – 1.95
Nasal polyps diagnosis			0.03<br>Chi-square	
No	8270 (91.6)	761 (8.4)		
Yes	8 (72.7)	3 (27.3)		4.08<br>1.08 – 15.39
Anaphylaxis diagnosis			0.54<br>Chi-square	
No	8274 (91.5)	764 (8.5)		
Yes	4 (100)	0 (0)		
Beta-blocker prescription*			0.32<br>Chi-square	
No	7097 (91.7)	645 (8.3)		
Yes	1181 (90.8)	119 (9.2)		1.11<br>0.90 – 1.36
Paracetamol prescription*			0.03<br>Chi-square	
No	4584 (92.1)	391 (7.9)		

Yes	3694 (90.8)	373 (9.2)		1.18<br>1.02 – 1.37
Statin prescription*			0.78<br>Chi-square	
No	4638 (91.6)	424 (8.4)		
Yes	3640 (91.5)	340 (8.5)		1.02<br>0.88 – 1.19
NSAID prescription*			0.17<br>Chi-square	
No	4485 (91.9)	394 (8.1)		
Yes	3793 (91.1)	370 (8.9)		1.11<br>0.96 – 1.29
Influenza vaccination			0.11<br>Chi-square	
No	2305 (92.8)	180 (7.2)		
Yes	5973 (91.1)	584 (8.9)		1.25<br>1.05 – 1.49
Pneumococcal vaccination			<0.01<br>Chi-square	
No	5394 (92.5)	440 (7.5)		
Yes	2884 (89.9)	324 (10.1)		1.38<br>1.19 – 1.60
Eosinophil count, mean ± SD	0.36 ± 0.7	0.36 ± 0.7	0.98	0.00<br>-0.06 – 0.06
Number of primary care respiratory consultations*, mean ± SD	3.39 ± 3.0	4.44 ± 4.4	<0.01	- 1.05<br>-1.29 – - 0.82
Number of all primary care consultations*, mean ± SD	14.40 ± 9.6	17.56 ± 13.1	<0.01	- 3.16<br>-3.89 - -2.43
Number of ED attendances for respiratory complaint*, mean ± SD	0.01 ± 0.13	0.02 ± 0.16	0.22	-0.01<br>-0.02 – 0.00
Number of all ED attendances*, mean ± SD	0.17 ± 0.55	0.17 ± 0.58	0.96	0.00<br>-0.04 – 0.04
Number of respiratory inpatient admissions*, mean ± SD	0.03 ± 0.18	0.03 ± 0.19	0.75	0.00<br>-0.02 – 0.01
Number of all inpatient admissions*, mean ± SD	0.28 ± 0.73	0.27 ± 0.70	0.65	0.03<br>-0.04 – 0.07
Number of respiratory OP attendances*, mean ± SD	0.09 ± 0.4	0.09 ± 0.5	0.86	0.00<br>-0.04 – 0.03

Number of all OP attendances*, mean ± SD	0.16 ± 0.6	0.16 ± 0.6	0.87	0.00<br>-0.05 – 0.04
Number of exacerbations*, mean ± SD	1.01 ± 1.4	1.11 ± 1.5	0.06	-0.10<br>-0.20 – - 0.01
Number of OCS courses*, mean ± SD	0.98 ± 1.4	1.08 ± 1.4	0.05	- 0.10<br>-0.20 – 0.00
FEV1 value (L), mean ± SD	1.44 ± 0.61	1.42 ± 0.60	0.38	0.02<br>-0.03 – 0.07
FEV1 / FVC ratio, mean ± SD	0.60 ± 0.5	0.59 ± 0.1	0.66	0.01<br>-0.03 – 0.05
Count of SABA prescriptions*, mean ± SD	3.99 ± 5.1	4.15 ± 5.5	0.41	-0.162<br>-0.54 – - 0.22
Count of ICS inhalers*, mean ± SD	5.87 ± 6.2	6.28 ± 6.6	0.08	-0.41<br>-0.87 – 0.05
Count of LAMA prescriptions*, mean ± SD	3.23 ± 4.6	3.57 ± 4.8	0.60	-0.33<br>-0.67 – - 0.01
Count of LABA inhalers*, mean ± SD	0.40 ± 2.1	0.60 ± 2.6	0.02	-0.20<br>-0.36 - -0.04
Count of theophylline prescriptions*, mean ± SD	0.48 ± 2.5	0.43 ± 2.2	0.55	0.06<br>-0.13 – 0.24
Count of LTRA prescriptions*, mean ± SD	0.12 ± 1.1	0.19 ± 1.3	0.06	0.04<br>-0.16 – 0.00
Count of SAMA prescriptions*, mean ± SD	0.91 ± 2.9	0.81 ± 2.7	0.32	0.10<br>-0.09 – 0.31
Index duration			0.43<br>Chi-square	
7 days	6284 (91.8)	564 (8.2)		
< 7 days	858 (90.8)	87 (9.2)	0.31<br>Chi-square	1.13<br>0.89 – 1.43
> 7 days	1136 (91.0)	113 (9.0)	0.34<br>Chi-square	1.11<br>0.90 – 1.37
Index antibiotic			<0.01<br>Chi-square	
Amoxicillin	4914 (92.6)	395 (7.4)		
Not amoxicillin	3364 (90.1)	369 (9.9)		1.37<br>1.18 – 1.58
ICS treatment			0.49	

			Chi-square	
ICS	5245 (91.5)	486 (8.5)		1.00
Treatment not including ICS	1887 (91.2)	183 (8.8)	0.62<br>Chi-square	1.05<br>0.88 – 1.25
No treatment	1146 (92.3)	95 (7.7)	0.34<br>Chi-square	0.89<br>0.71 – 1.12

Supplementary table 2: Results of univariable analysis of all factors for repeat antibiotic prescription for all indications, according to type of data. Univariable p value by independent sample T-test unless otherwise stated. Columns are n (%), unless otherwise stated.

(OR: odds ratio; 95 % CI: 95 % confidence interval; * in year prior to index prescription)

Variable	**No antibiotic prescription within 14 days of index prescription N (%)**	**Further antibiotic prescription within 14 days of index prescription N (%)**	**p value**	**OR 95% CI**	**Mean difference 95% CI**
Gender			0.99<br>Chi-square		
Female	3666 (84.4)	677 (15.6)			
Male	3946 (84.4)	729 (15.6)		1.00<br>0.89 – 1.12	
Age, mean ± SD	70.46 ± 10.6	70.97 ± 10.8	0.10		-0.511<br>-1.12 – 0.093
BMI, mean ± SD	27.35 ± 6.1	27.22 ± 6.0	0.47		0.13<br>-0.22 – 0.48
Smoking status			0.05<br>Chi-square		
Non-smoker	664 (84.4)	123 (15.6)	0.55<br>Chi-square	0.94<br>0.76 – 1.15	
Current smoker	2764 (85.5)	468 (14.5)	0.02<br>Chi-square	0.86<br>0.76 – 0.97	
Ex-smoker	4058 (83.5)	801 (16.5)		1.00	
Location			0.36<br>Chi-square		
Midlands and East	3728 (83.9)	713 (16.1)			
London	55 (84.6)	10 (15.4)	0.88<br>Chi-square	0.95<br>0.48 – 1.87	
North	1508 (85.8)	250 (14.2)	0.07<br>Chi-square	0.87<br>0.74 – 1.01	
South	1771 (84.5)	325 (15.5)	0.57	0.96	

			Chi-square	0.83 – 1.11	
Recorded comorbidity					
Eczema diagnosis			0.35<br>Chi-square		
No	5693 (84.2)	1068 (15.8)			
Yes	1919 (85.0)	338 (15.0)		0.94<br>0.82 – 1.07	
Rhinitis diagnosis			0.61<br>Chi-square		
No	6691 (84.5)	1229 (15.5)			
Yes	921 (83.9)	177 (16.1)		1.05<br>0.88 – 1.24	
Diabetes diagnosis			0.67<br>Chi-square		
No	6492 (84.5)	1193 (15.5)			
Yes	1120 (84.0)	213 (16.0)		1.04<br>0.88 – 1.21	
Gastro-oesophageal reflux disease diagnosis			0.04<br>Chi-square		
No	7424 (84.5)	1358 (15.5)			
Yes	188 (79.7)	48 (20.3)		1.40<br>1.01 – 1.93	
Anxiety / depression diagnosis			0.63<br>Chi-square		
No	6970 (84.5)	1282 (15.5)			
Yes	642 (83.8)	124 (16.2)		1.05<br>0.86 – 1.28	
Cardiovascular disease diagnosis			<0.01<br>Chi-square		
No	6508 (85.1)	1141 (14.9)			
Yes	1104 (80.6)	265 (19.4)		1.37<br>1.18 – 1.59	
Nasal polyps diagnosis			0.29<br>Chi-square		
No	7604 (84.4)	1403 (15.6)			
Yes	8 (72.7)	3 (27.2)		2.03<br>0.54 – 7.67	
Anaphylaxis diagnosis			0.39<br>Chi-square		
No	7608 (84.4)	1406 (15.6)			
Yes	4 (100)	0 (0)		NA	
Beta-blocker prescription*			0.12<br>Chi-square		
No	6535 (84.7)	1185 (15.3)			
Yes	1077 (83.0)	221 (17.0)		1.13<br>0.97 – 1.33	
Paracetamol prescription*			<0.01<br>Chi-square		
No	4243 (85.5)	720 (14.5)			

Yes	3369 (83.1)	686 (16.9)		1.20<br>1.07 – 1.35	
Statin prescription*			0.82<br>Chi-square		
No	4258 (84.3)	791 (15.7)			
Yes	3354 (84.5)	615 (15.5)		0.99<br>0.88 – 1.11	
NSAID prescription*			0.04<br>Chi-square		
No	4144 (85.1)	724 (14.9)			
Yes	3468 (83.6)	682 (16.4)		1.13<br>1.00 – 1.26	
Influenza vaccination			0.08<br>Chi-square		
No	2116 (85.5)	359 (14.5)			
Yes	5496 (84.0)	1047 (16.0)		1.121<br>0.99 – 1.28	
Pneumococcal vaccination			0.08<br>Chi-square		
No	4938 (84.9)	878 (15.1)			
Yes	2674 (83.5)	528 (16.5)		1.11<br>0.99 – 1.25	
Eosinophil count, mean ± SD	0.36 ± 0.69	0.36 ± 0.70	0.90		0.00<br>-0.04 – 0.05
Number of primary care respiratory consultations*, mean ± SD	3.40 ± 3.02	3.86 ± 3.67	<0.01		-0.46<br>-0.63 - - 0.28
Number of all primary care consultations*, mean ± SD	14.31 ± 9.44	16.57 ± 11.88	<0.01		-2.26<br>-2.82 - - 1.70
Number of ED attendances for respiratory complaint*, mean ± SD	0.01 ± 0.13	0.02 ± 0.15	0.10		-0.01<br>-0.01 – 0.00
Number of all ED attendances*, mean ± SD	0.16 ± 0.53	0.19 ± 0.62	0.06		-0.03<br>-0.06 – 0.00
Number of resp inpatient admissions*, mean ± SD	0.03 ± 0.18	0.03 ± 0.19	0.48		0.00<br>-0.01 – 0.01
Number of all inpatient admissions*, mean ± SD	0.27 ± 0.71	0.31 ± 0.77	0.12		-0.03<br>-0.07 – 0.01
Number of respiratory OP	0.08 ± 0.42	0.12 ± 0.51	<0.01		-0.04<br>-0.06 - - 0.01

attendances*, mean ± SD					
Number of all OP attendances*, mean ± SD	0.15 ± 0.56	0.19 ± 0.61	0.02		-0.04<br>-0.07 - - 0.01
Number of exacerbations*, mean ± SD	0.99 ± 1.35	1.18 ± 1.57	<0.01		-0.20<br>-0.28 - - 0.12
Number of OCS courses*, mean ± SD	0.96 ± 1.34	1.16 ± 1.56	<0.01		-0.20<br>-0.28 - - 0.12
FEV1 value (L), mean ± SD	1.44 ± 0.61	1.40 ± 0.59	0.01		0.05<br>0.01 – 0.08
FEV1 / FVC ratio, mean ± SD	0.60 ± 0.53	0.59 ± 0.16	0.58		0.01<br>-0.02 – 0.04
Count of SABA prescriptions*, mean ± SD	3.96 ± 5.13	4.26 ± 5.33	0.04		-0.30<br>-0.60 - - 0.01
Count of ICS inhalers*, mean ± SD	5.76 ± 6.11	6.72 ± 6.67	<0.01		-0.97<br>-1.32 - - 0.61
LAMA inhalers*	3.79 ± 5.42	4.60 ± 5.84	<0.01		-0.81<br>-1.13- -0.50
LABA inhalers*	0.42 ± 2.19	0.45 ± 2.18	0.54		-0.04<br>-0.16 – 0.09
Theophylline prescriptions*	0.45 ± 2.44	0.63 ± 2.59	0.01		-0.18<br>-0.32 - - 0.04
LTRA prescriptions*	0.12 ± 1.11	0.12 ± 0.97	0.96		0.00<br>-0.06 – 0.06
SAMA prescriptions*	0.92 ± 2.87	0.84 ± 2.69	0.32		0.08<br>-0.08 – 0.24
Index antibiotic duration			0.86<br>Chi-square		
7 days	5757 (84.3)	1072 (15.7)		1.00	
< 7 days	801 (84.9)	142 (15.1)	0.61<br>Chi-square	0.95<br>0.79 – 1.15	
> 7 days	1054 (84.6)	192 (15.4)	0.80<br>Chi-square	0.98<br>0.83 – 1.16	
Index antibiotic			<0.01<br>Chi-square		
Amoxicillin	4548 (85.8)	751 (14.2)			
Not amoxicillin	3064 (82.4)	655 (17.6)		1.30<br>1.16 – 1.45	
ICS treatment			<0.01<br>Chi-square		
ICS	4762 (83.3)	958 (16.7)			

Treatment not including ICS	1766 (85.6)	296 (14.4)	0.01<br>Chi-square	0.83<br>0.72 – 0.96	
No treatment	1084 (87.7)	152 (12.3)	<0.01<br>Chi-square	0.70<br>0.58 – 0.84	

Supplementary table 1: Results of clinically and statistically significant uni- and multivariable analysis for repeat antibiotic prescription for any indication within 14 days of index duration, according to type of data.

(OR: odds ratio; 95 % CI: 95 % confidence interval; * in year prior to index prescription). Blank entries relate to variables not included in the multivariable model.

	**Univariable analysis results**			**Multiple logistic regression model results**	
**Variable**	**OR<br>95 % CI**	**Mean difference<br>95% CI**	**p value**	**OR<br>95 % CI**	**p value**
**Age**		-0.51<br>-1.12 – 0.09	0.10		
**Smoking status**			0.05		
**Ex-smoker**	1.00				
**Current smoker**	0.86<br>0.76 – 0.97		0.02		
**Non-smoker**	0.94<br>0.76 – 1.15		0.55		
**Gastro-oesophageal reflux disease diagnosis**	1.40<br>1.01 – 1.93		0.04		
**Cardiovascular disease diagnosis**	1.40<br>1.18 – 1.59		<0.01	1.23<br>1.05 – 1.45	0.01
**Paracetamol prescription***	1.20<br>1.07 – 1.35		<0.01		
**NSAID prescription***	1.13<br>1.00 – 1.26		0.04		
**Influenza vaccination**	1.12<br>0.99 – 1.28		0.08		
**Pneumococcal vaccination**	1.11<br>0.99 – 1.25		0.08		
**Number of primary care respiratory consultations***		-0.46<br>-0.63 - - 0.28	<0.01		
**Number of all primary care consultations***		-2.26<br>-2.82 - - 1.70	<0.01	1.02<br>1.01 – 1.02	<0.01
**Number of ED attendances for respiratory complaint***		-0.01<br>-0.01 – 0.00	0.10		
**Number of all ED attendances***		-0.03<br>-0.06 – 0.00	0.06		
**Number of respiratory OP appointments***		-0.034<br>-0.06 - - 0.01	<0.01		

**Number of all OP appointments***		-0.04<br>-0.07 - - 0.01	0.02		
**Number of exacerbations***		-0.20<br>-0.28 - - 0.12	<0.01	1.04<br>1.00 – 1.09	0.06
**Number of OCS***		-0.20<br>-0.28 - - 0.12	<0.01		
**FEV1 value (L)**		0.05<br>0.01 – 0.08	0.01		
**Number of SABA prescriptions***		-0.30<br>-0.60 - - 0.01	0.04		
**Number of ICS inhalers***		-0.97<br>-1.32 - - 0.61	<0.01	1.01<br>1.00 – 1.03	0.01
**Number of LAMA inhalers***		-0.81<br>-1.13 - -0.50	<0.01	1.01<br>1.00 – 1.03	0.01
**Number of Theophylline prescriptions***		-0.18<br>-0.32 - - 0.04	0.01		
**Index antibiotic not amoxicillin**	1.30<br>1.16 – 1.45		<0.01<br>Chi-square	1.20<br>1.06 – 1.35	0.00
**ICS treatment**			<0.01<br>Chi-square		
**ICS**	Index				
**No ICS**	0.83<br>0.72 – 0.96		0.01<br>Chi-square		
**No treatment**	0.70<br>0.58 – 0.84		<0.01<br>Chi-square		

Supplementary table 4: Comparison of effects of clinically plausible interaction terms on final multiple logistic regression model for repeat LRTI antibiotic prescriptions.

	**AUC**	**95 % CI**	**Cox-Snell R^2**
**Original model**	0.613	0.59 – 0.63	0.013
**Original model with interaction term:**			
Number of all primary care* and respiratory consultations*	0.613	0.59 – 0.63	0.013
Age and smoking status	0.613	0.59 – 0.63	0.013
Age and influenza vaccination	0.613	0.59 – 0.63	0.013
Age and pneumococcal vaccination	0.613	0.59 – 0.63	0.013
Age and number of primary care respiratory consultations*	0.613	0.59 – 0.63	0.013
Age and number of all primary care consultations*	0.613	0.59 – 0.63	0.013

Number of primary care respiratory consultations* and pneumococcal vaccination	0.613	0.59 – 0.63	0.013
Number of all primary care consultations* and pneumococcal vaccination	0.613	0.59 – 0.63	0.013

(AUC: area under curve; 95 % CI: 95 % confidence interval; * in year prior to index prescription)

Supplementary table 5: Comparison of effects of clinically plausible interaction terms on final multiple logistic regression model for predicting repeat prescription of all antibiotics. (* in year prior to index prescription)

	**AUC**	**95 % CI**	**Cox-Snell R^2**
**Original model**	0.580	0.56 – 0.60	0.012
**Original model with interaction term:**			
Number of all primary care* and respiratory consultations*	0.580	0.56 – 0.60	0.012
Age and smoking status	0.580	0.56 – 0.60	0.012
Age and influenza vaccination	0.580	0.56 – 0.60	0.012
Age and pneumococcal vaccination	0.580	0.56 – 0.60	0.012
Age and number of primary care respiratory consultations*	0.580	0.56 – 0.60	0.012
Age and number of all primary care consultations*	0.580	0.56 – 0.60	0.012
Number of primary care respiratory consultations* and pneumococcal vaccination	0.580	0.56 – 0.60	0.012
Number of all primary care consultations* and pneumococcal vaccination	0.580	0.56 – 0.60	0.012

Supplementary table 6: Used Read code list for LRTI.

**Read Code**	**Term**
H06z0	Chest infection NOS
XE0Xs	Chest infection NOS
H060.	Acute bronchitis
XE0Xr	Acute bronchitis
H06z1	Lower resp tract infection
X1004	Infection of lower respiratory tract
XE0Xt	Acute lower respiratory tract infection
H30..	Bronchitis unspecified
H062.	Acute lower respiratory tract infection
H30z.	Bronchitis NOS
XE0YL	Bronchitis unspecified
H302.	Wheezy bronchitis
XaDtP	Bronchitis
XM1QX	Acute wheezy bronchitis
H060z	Acute bronchitis NOS
X1006	Chest infection - unspecified bronchitis
H3122	Acute exacerbation of chronic obstructive airways disease
X101i	Unspecified

Xa35I	Unspecified
H06..87	Acute bronchitis and bronchiolitis

Supplementary figure 1: Treemap chart of antibiotic duration and drug of repeat prescriptions for all indications. Absolute numbers presented. a: amoxicillin; b: ery-/clarithromycin; c: doxycycline; d: co-amoxiclav; e: other. Missing data due to missing duration (304 cases).

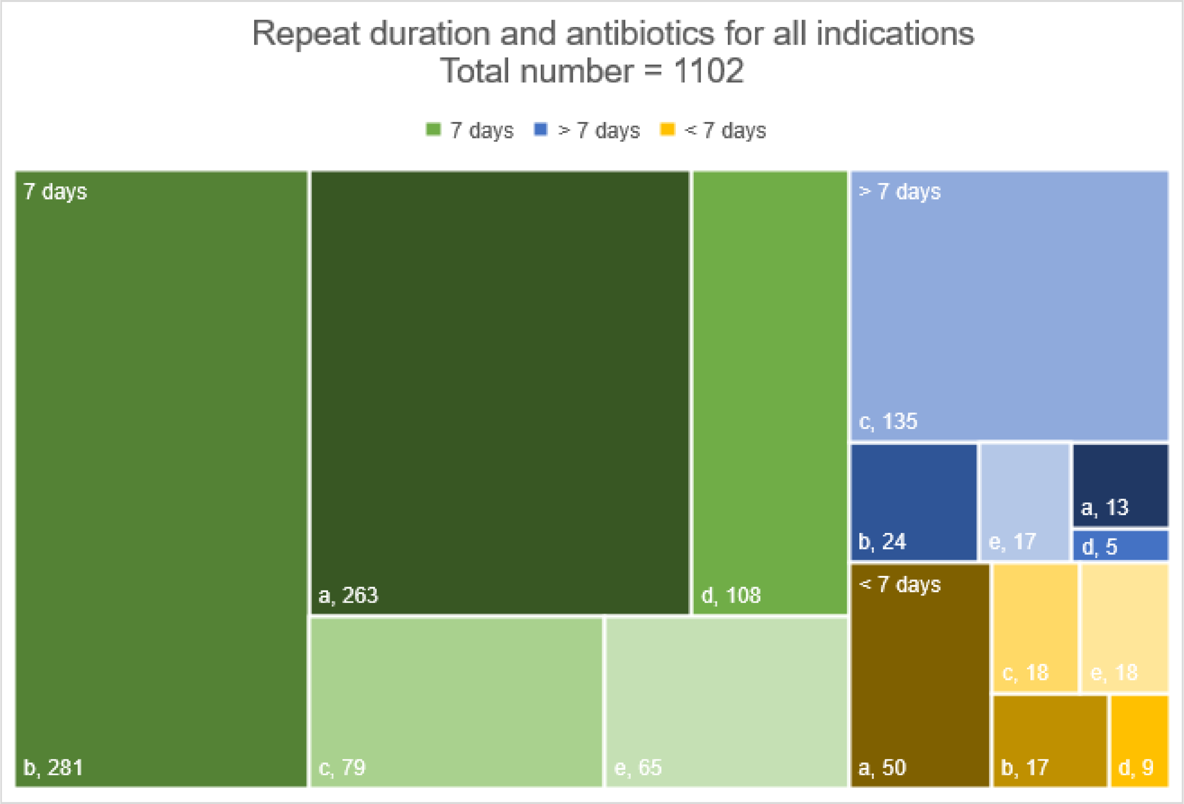

Supplement: Supplementary data [file bmjresp-2019-000458supp001.pdf]
